# Efficacy of horizontal muscle augmentation combined inferior oblique muscle shortening for pediatric strabismus: Study Protocol

**DOI:** 10.1097/MD.0000000000017941

**Published:** 2019-11-15

**Authors:** Xiu-Mei Du

**Affiliations:** Department of Ophthalmology, Xi’an No. 4 Hospital, Xi’an, China.

**Keywords:** efficacy, horizontal muscle augmentation, inferior oblique muscle shortening, pediatric strabismus, safety

## Abstract

**Background:**

This study will explore the efficacy and safety of horizontal muscle augmentation (HMA) combined inferior oblique muscle shortening (IOMS) for the treatment of pediatric strabismus (PS).

**Methods:**

Literature search for studies will be carried out in the following databases: Cochrane Library, MEDILINE, EMBASE, CINAHL, Web of Science, PsycINFO, CBM, and CNKI. We will search all these databases without language and publication status restrictions. Two independent authors will perform selection of studies, data collection and management, risk of bias evaluation. A third author will be consulted with the help of discrepancies.

**Results:**

This study will provide a synthesis of existed evidence for HMA combined IOMS for the treatment of PS.

**Conclusion:**

The results of this study will provide evidence to evaluate the efficacy and safety of HMA combined IOMS for the treatment of PS, which can help to guide clinical decision-making.

**Systematic review registration:**

PROSPERO CRD42019149716.

## 1 Introduction

Pediatric strabismus (PS) is misalignment disorder of the eyes among children population.^[1–3]^ It has been reported that about 3% children can experience such disorder.^[4]^ If it is untreated, about 50% children with PS may cause visual loss due to the amblyopia.^[4–6]^ Several factors are supposed to response for PS, such as refractive error, muscle imbalance, retinoblastoma, or other serious ocular defects or diseases.^[7–9]^ Several managements can help to treat PS, including patching or atropine drops, contact lenses or eyeglasses, eye exercises, and surgical alignments (such as horizontal muscle augmentation (HMA), inferior oblique muscle shortening (IOMS)).^[10–21]^ Previous studies have reported that HMA and IOMS can benefit children with PS.^[22–31]^ However, no studies have addressed this topic systematically. Thus, this study will systematically assess the efficacy and safety of HMA and IOMS for the treatment of PS.

## 2 Methods

### 2.1 Dissemination and ethics

This study is a literature-based study; therefore, no ethical approval is required. This study is expected to be published at a peer-reviewed journal.

### 2.2 Study registration

This study protocol has been registered on PROSPERO CRD42019149716. We report this study based on the Cochrane Handbook for Systematic Reviews of Interventions and the Preferred Reporting Items for Systematic Reviews and Meta-Analysis Protocol statement guidelines.^[32]^

### 2.3 Inclusion and exclusion criteria

#### 2.3.1 Types of studies

Any randomized controlled trials (RCTs) of HMA combined IOMS for the treatment of PS. We will exclude non-RCTs and quasi-RCTs.

#### 2.3.2 Types of participants

Participants diagnosed with PS will be included without restrictions of ethnicity, gender, and age.

#### 2.3.3 Types of interventions

The experimental group has used HMA combined IOMS for the treatment.

The control group has received any interventions, except the single therapy of HMA, IOMS, or combination of HMA and IOMS.

#### 2.3.4 Types of outcome measurements

The primary outcome includes the incidence of postoperative nausea and vomiting. The secondary outcomes consist of pain intensity (as measured by any pain scores), incidence of occulocardiac reflex, incidence of wound healing, incidence of wound infection, quality of life (as measured by any related scales), and adverse events.

### 2.4 Search strategy

#### 2.4.1 Electronic databases searching

We will systematically search Cochrane Library, MEDILINE, EMBASE, CINAHL, Web of Science, PsycINFO, CBM, and CNKI without language, time of publication, and publication status restrictions. All electronic databases will be searched from inceptions to the present. A detailed search strategy for Cochrane Library will be showed in Table 1. We will also build identical search strategy to the other electronic databases.

**Table 1:**
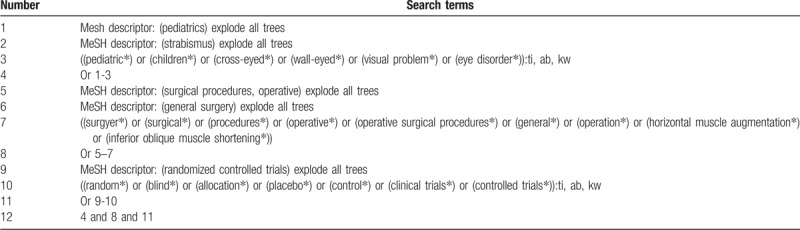
Search strategy for Cochrane Library database.

#### 2.4.2 Other resources searching

We will also search dissertations, ongoing studies, clinical registry, and reference list of qualified studies to avoid missing any potential studies.

### 2.5 Data synthesis and statistical analysis

#### 2.5.1 Study selection

Two authors will independently identify titles and abstracts of all literatures through the search strategy to check eligible studies. Any different opinions will be solved through discussion with a third author invited. We will exclude any irrelevant studies after initial screen, and full texts of remaining studies will be carefully read to further check their edibility criteria. The process of study selection will be presented in diagram chart.

#### 2.5.2 Data collection and management

We will use predefined data acquisition sheet to extract data from each eligible study by 2 independent authors. Any discrepancies noticed in the process of data collection will be solved via another author. The following extracted information includes study characteristics (such as time of publication, title, first author, et al); patient characteristics (such as sample size, gender, age, race, et al); study methods (such as randomization, blinding, allocation, et al); treatment details (such as duration, frequency, et al); outcomes (such as primary and secondary outcomes, et al); and safety.

#### 2.5.3 Dealing with missing data

When the essential missing information occurs, primary authors will be contacted to require that. We will analyze available data only when that information is not achievable.

#### 2.5.4 Risk of bias assessment

Two authors will independently evaluate the study quality by using Cochrane risk of bias. This tool includes selection bias, performance bias, detection bias, attrition bias, reporting bias, and other possible sources. Each one is further graded as low, unclear or high risk of bias. If there are inconsistent results occur between 2 authors, a third author will help to make final decision through discussion.

#### 2.5.5 Measurement of treatment effect

For dichotomous outcome data, we will utilize risk ratio along with its 95% confidence intervals, whereas continuous data will be calculated as mean difference or standardized mean difference along with 95% confidence intervals.

#### 2.5.6 Assessment of heterogeneity

Heterogeneity among included RCTs will be checked using *I*^2^ test. The study is considered as low heterogeneity if *I*^2^ ≤ 50%. However, when *I*^2^ > 50%, there is significant heterogeneity.

#### 2.5.7 Data synthesis

We will use RevMan 5.3 software to perform statistical analysis. We will use a fixed-effects model to pool the data if low heterogeneity occurs among included RCTs (*I*^2^ ≤ 50%). Meanwhile, a meta-analysis will be conducted based on the same treatments, and outcome measurements. We will apply a random-effects model to pool the data if substantial heterogeneity exists among eligible RCTs (*I*^2^ > 50%). At the same, we will perform subgroup analysis, and meta-regression test to explore the possible reasons for the high heterogeneity.

#### 2.5.8 Publication bias

We will use Funnel plot and Egger regression test to visually inspect reporting bias if a sufficient number of included RCTs (more than 10 studies) are available.^[33]^

#### 2.5.9 Subgroup analysis

We will employ subgroup analysis to explore the source of heterogeneity according to the different treatments, comparators, and outcomes.

#### 2.5.10 Sensitivity analysis

Sensitivity analysis will be carried out to evaluate the robustness and satiability of pooled outcomes according to the different methodological quality.

## 3 Discussion

Although previous studies have reported that HMA and IOMS can benefit patients with PS,^[22–31]^ there is still no systematic study to investigate its efficacy and safety for the treatment of patients with PS. Therefore, this study will firstly assess the efficacy and safety of HMA and IOMS for PS comprehensively and systematically. We will summarize the current evidence on the efficacy and safety of HMA and IOMS compared to other controls in the treatment of PS. The findings of this study will provide helpful evidence for both clinical practice and further researches.

## Author contributions

**Conceptualization:** Xiu-mei Du.

**Data curation:** Xiu-mei Du.

**Formal analysis:** Xiu-mei Du.

**Investigation:** Xiu-mei Du.

**Methodology:** Xiu-mei Du.

**Project administration:** Xiu-mei Du.

**Resources:** Xiu-mei Du.

**Software:** Xiu-mei Du.

**Supervision:** Xiu-mei Du.

**Validation:** Xiu-mei Du.

**Visualization:** Xiu-mei Du.

**Writing – original draft:** Xiu-mei Du.

**Writing – review & editing:** Xiu-mei Du.
